# Influence of conformity on the wear of total knee replacement: An experimental study

**DOI:** 10.1177/0954411917746433

**Published:** 2017-12-17

**Authors:** Claire L Brockett, Silvia Carbone, John Fisher, Louise M Jennings

**Affiliations:** Institute of Medical and Biological Engineering, School of Mechanical Engineering, University of Leeds, Leeds, UK

**Keywords:** Total knee replacement, polyethylene, wear, conformity

## Abstract

Wear of total knee replacement continues to be a significant factor influencing the clinical longevity of implants. Historically, failure due to delamination and fatigue directed design towards more conforming inserts to reduce contact stress. As new generations of more oxidatively stable polyethylene have been developed, more flexibility in bearing design has been introduced. The aim of this study was to investigate the effect of insert conformity on the wear performance of a fixed bearing total knee replacement through experimental simulation. Two geometries of insert were studied under standard gait conditions. There was a significant reduction in wear with reducing implant conformity. This study has demonstrated that bearing conformity has a significant impact on the wear performance of a fixed bearing total knee replacement, providing opportunities to improve clinical performance through enhanced material and design selection.

## Introduction

Total knee replacement (TKR) is an effective treatment option for end-stage knee arthritis, reducing pain and improving joint function for millions of patients worldwide, with 10-year survivorship reported above 90% in several countries.^1–4^ In recent years, the demand for TKR has increased globally, with a 600% increase in TKR predicted in the United States by 2035, with respect to implantation rates in 2007.^5^ More recently, factors such as changing demographics and body mass index have been considered to more significantly impact on projected TKR demand, compared with total hip replacement. As the mean average life expectancy increases, patients will rely on their TKRs for an increasing length of time, therefore improving longevity and reducing the need for revision has become a pressing need.

Polyethylene wear continues to be a major contributing factor to the longevity of TKRs, with loosening and wear frequently highlighted as reasons for revision clinically.^6,7^ Many factors influence the wear performance of a TKR, including design, material, patient activity levels and surgical alignment.

In the last decade, significant advances have been made in material properties, with the introduction of cross-linked ultra-high-molecular-weight polyethylene (UHMWPE) to reduce volumetric wear rates. Experimental studies have indicated improved wear performance compared with conventional UHMWPE,^8–10^ and clinical studies have indicated a comparable or improved performance.^4,11^ However, a trade-off between wear resistance associated with the level of cross-linking, and the mechanical properties of the material has been highlighted.^12,13^ Asano et al.^14^ demonstrated an almost linear relationship between wear resistance and radiation dose, highlighting a corresponding reduction in mechanical properties. More recently, Attwood et al.^13^ demonstrated that an increase in cross-linking was associated with a decrease in resistance to fatigue crack propagation. The complexity of the motion occurring on the polyethylene surface of a fixed bearing TKR, with large rotational and displacement motions, means that it may be susceptible to failure through fatigue. The application of moderately cross-linked UHMWPE in TKR designs permits improved wear resistance while maintaining mechanical properties.

Clinical failures associated with mechanical fracture and delamination of earlier generation, UHMWPE made the conformity of a knee replacement a key design consideration for reducing this mode of failure.^15,16^ There was a general trend towards more conforming, and lower stress, designs to mitigate the risk of fatigue and mechanical failure. However, biomechanical analysis has suggested that these highly conforming knee replacements may cause over-constraint of the knee joint during normal daily activities.^17,18^ A retrieval study comparing a conforming and less conforming knee replacement design manufactured from a modern polyethylene highlighted that the higher conformity inserts had greater surface damage through third-body debris trapped within the dished surface, and were more susceptible to fatigue wear and delamination due to secondary stresses generated from the constraints of the joint.^19^

Improvements in polyethylene processing and sterilisation have enhanced the mechanical properties of UHMWPE^20^ and therefore increased the opportunities available to explore different knee replacement geometry. Previous studies have proposed a new wear law where the wear volume is independent of contact pressure, instead correlates with contact area, thus suggesting that a lower conformity insert would lead to lower volumetric wear due to the lower contact area.^21^ The study compared the wear performance of a fixed bearing TKR with a conventional polyethylene insert, in current clinical use, with a custom-made flat insert of the same material. It demonstrated significantly lower wear rates for the flat insert and showed smaller wear scar areas.^21^

The aim of this study was to further investigate the influence of conformity on the wear performance of a moderately cross-linked polyethylene fixed bearing TKR through different design configurations.

## Materials

The wear of the fixed bearing TKR was investigated using two different insert geometries, a lipped insert of current clinical design and a custom-made flat insert with an equivalent minimum thickness to the clinical product (10 mm). The lipped inserts had an equivalent minimum thickness to the curved inserts reported in a previous study.^21^ The inserts were less conforming than those previously reported; in the medial–lateral direction the radii were comparable, however, the radii of the lipped inserts were approximately double those of the curved inserts previously studied in the anterior–posterior direction.^21^ The custom-made flat inserts were manufactured from thicker ‘off-the-shelf’ lipped inserts that were CNC-machined flat to give a minimum thickness of 10 mm. The fixed bearing knee replacement used in this study was the Sigma Cruciate Retaining design (DePuy Synthes, UK). The femoral bearings were manufactured from a cobalt-chromium-molybdenum (CoCrMo) alloy, and the polyethylene inserts are clipped into a highly polished CoCrMo tibial tray. Tests were conducted with a moderately cross-linked GUR1020 UHMWPE (5MRad irradiated and remelted) insert (XLK^TM^ material).

## Methods

**Figure 1.**
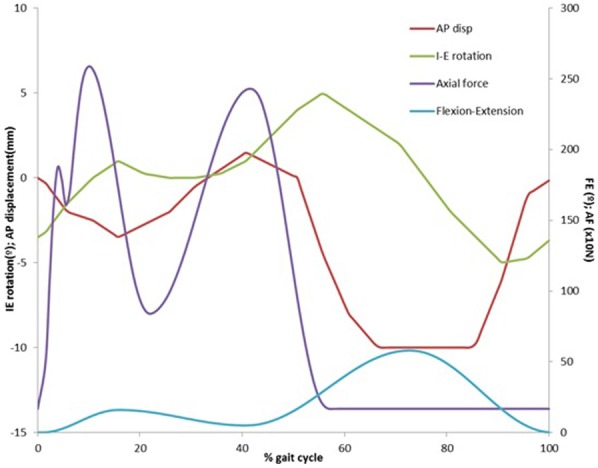
Input conditions for wear simulation.

The experimental studies were conducted using the Leeds ProSim pneumatic six station knee simulator,^22^ under displacement control (Simulator Solutions, UK). Each station had 6 degrees of freedom, of which 4 were driven – axial load, flexion–extension, tibial rotation and tibial anterior–posterior displacement. The femoral axis loading (peak load of 2.6 kN) and flexion–extension (0°–58°) input profiles were taken from ISO 14243-3^23^ for all studies (Figure 1).

**Figure 2.**
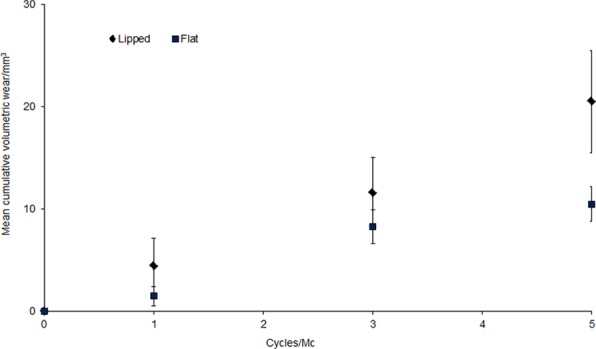
Cumulative mean wear rates for insert geometries (±95% confidence limits).

The I/E tibial rotation was ±5° based on the natural kinematics of the knee as described by Lafortune et al.^24^ Anterior–posterior translation was displacement controlled for all studies. This study examines cruciate-retaining implants, which rely in vivo on the natural tissues and thus have no intrinsic stability provided by the implant geometry. Therefore, displacement controlled studies were performed to replicate the constraint provided by the soft tissue in vivo.^22^ A high kinematic condition was used for all studies, with an anteroposterior (AP) displacement of 0–10 mm (Figure 2).^25^ Abduction/adduction was allowed but not controlled. Six sets of bearings were tested for each design, mounted anatomically in each station. The central axis of the implant was offset from the axis of applied load from the centre of the joint by 7% of its width, in accordance with ISO 14243-1, to replicate a right knee. In order to eliminate station-specific differences, the samples were moved around the stations every million cycles.^22^ A comparison between demand inputs and simulator outputs for this simulator has previously been reported.^22^ The kinematics and loading profile for this study were monitored throughout and were comparable to the variation previously reported.

Each study was conducted for a period of five million cycles (Mc). The simulator was run at a frequency of 1 Hz. The lubricant used was 25% newborn calf serum, supplemented with 0.03% (v/v) sodium azide to retard bacterial growth and was changed every 0.33 Mc. The approximate protein concentration was 17 g/L. Prior to testing, all inserts were soaked in deionised water for a period of 4 weeks.^8^ This allowed for the inserts to reach equilibrium of absorption prior to the start of the study. Wear was determined gravimetrically through measurements of the inserts following the 4-week soak period, and at one, three and five million cycles during the study. At each measurement interval, the inserts were cleaned and left to dry for a period of 48 h in a controlled environment (temperature (20 °C±1 °C) and humidity (45% ± 5%)) prior to weighing. A Mettler AT201 (Mettler-Toledo, USA) digital microbalance, which had a resolution of 0.01 mg, was used for weighing the bearing inserts. The volumetric wear was calculated from the weight loss measurements, using a density of 0.934 mg/mm^3^, and unloaded soak controls were used to compensate for moisture uptake. The control samples were soaked in 25% bovine serum, as prepared for the test samples, and this serum was also changed every 0.33 Mc.

Digital images of the wear scars on the inserts at the completion of the study were obtained by manually tracing the outline of the wear scars on the superior surface of each insert and capturing the image on a Kodak DX6490 digital camera. The mean wear scar area, expressed as a percentage of overall superior surface area, was measured from the digital images using Image Pro Plus (Media Cybernetics, USA). Statistical analysis was performed using an independent two-sample t-test.

## Results

**Figure 3.**
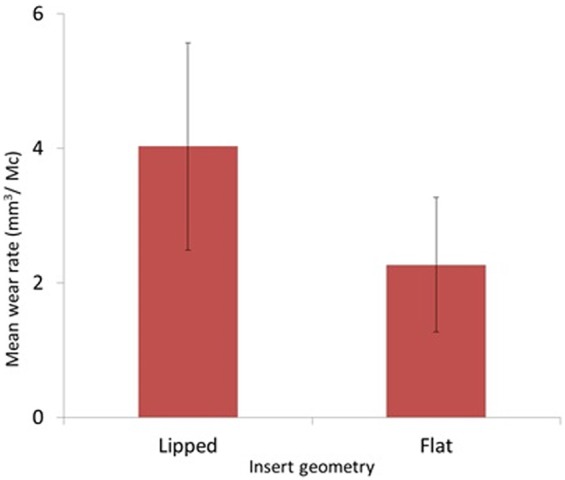
Mean wear rates for insert geometries (±95% confidence limits).

The cumulative wear and mean wear rates, with 95% confidence limits, for each bearing geometry are shown in Figures 2 and 3, respectively. The mean wear rate for the lipped inserts was 4.0 ± 1.0 mm^3^/Mc. In contrast, the mean wear rate for the custom-flat inserts was 2.3 ± 0.3 mm^3^/Mc. The wear rate for the flat inserts was significantly lower than the lipped inserts under these test conditions (p < 0.05). The mean difference between lipped and flat inserts was 49%.

**Figure 4.**
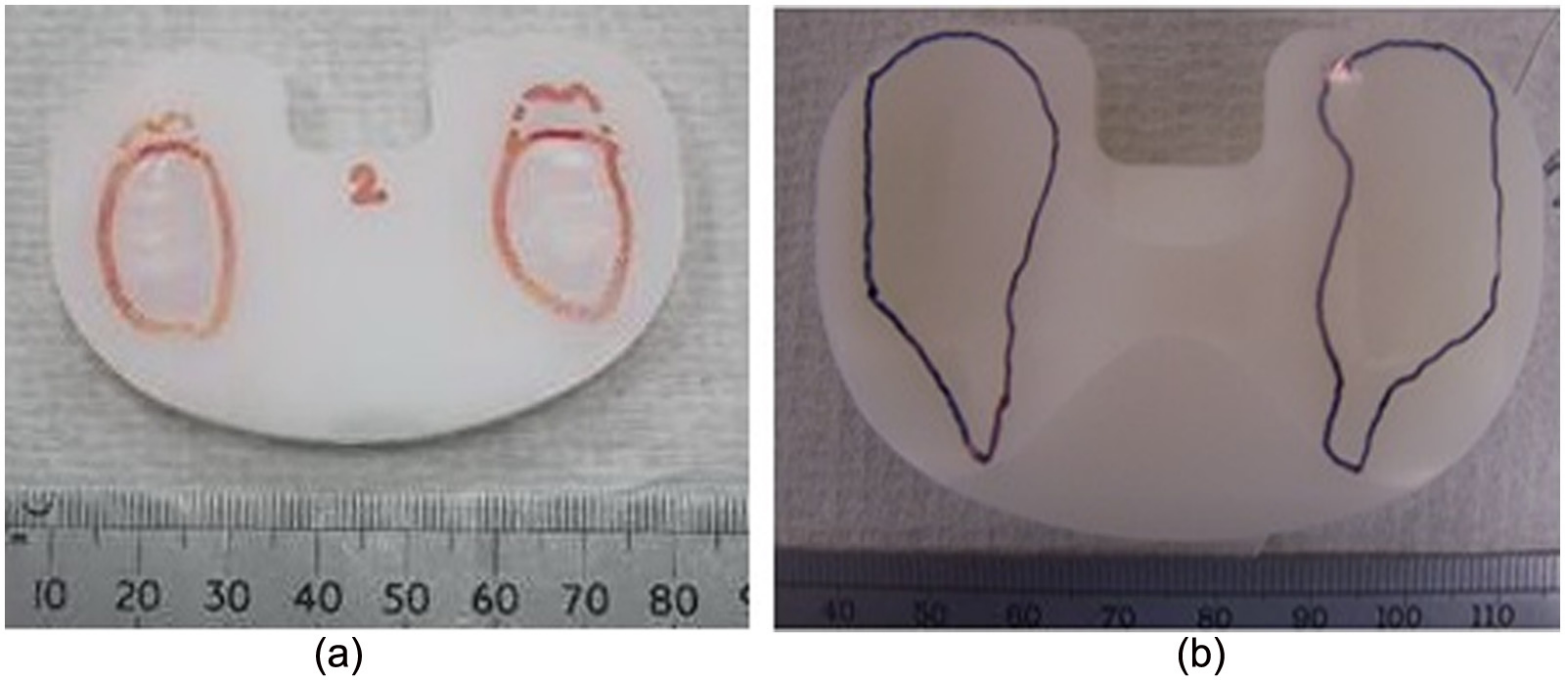
Examples of final wear scars for each design: (a) flat insert and (b) lipped insert.

The mean wear scar area, expressed as a percentage of overall superior surface area, was 29% ± 4% for the lipped inserts and 10% ± 2% for the flat inserts (Figure 4). The wear scar areas on the flat inserts were smaller than the lipped inserts, and specifically were shorter in the anterior–posterior direction. The data associated with this paper are openly available from the University of Leeds Data Repository.^26^

## Discussion

Enhanced mechanical properties of the polyethylene through advanced manufacturing and sterilisation processes have enabled consideration of lower conformity implants with higher contact pressures to reduce surface wear.^20^ The influence of bearing conformity and material on the wear performance of TKRs was investigated through a series of experimental knee wear studies. The influence of conformity was explored with an insert design in current clinical use and a bespoke flat insert articulating with a clinically relevant femoral bearing.

The effect of conformity on the wear of fixed bearing knee replacements was demonstrated through a knee wear simulator study. The highest wear rates were observed in the most conforming bearings. There was a significant reduction in wear observed as the conformity reduced. Previous studies undertaken within the same research group have explored conventional polyethylene and more conforming inserts under comparable test conditions.^8,21^ When these data are combined with this study, the trend for decreasing wear rate with decreasing conformity is more apparent.

The trend for decreasing wear with decreasing conformity was also reflected in the size of the wear scar areas, with the smallest wear scars observed on the flat inserts. Much of the literature associated with the wear response of polyethylene to contact area and pressure conditions has been generated through simple geometry pin-on-plate and pin-on-disk studies^27–29^ While these methods are useful, enabling a parametric approach to investigating the effect of conditions, such as cross-shear and contact pressure on wear, it is important to recognise the limitations of such studies in replicating relevant conditions for total joint replacement. In order to fully assess the implication of design changes on a TKR, full joint simulation or modelling is required.

A previous study highlighted the potential for decreasing wear rate with increasing contact stress.^21^ More recently, computational modelling has demonstrated the wear patterns for the different levels of conformity alter such that the cross-shear ratio at the periphery of the contact area is increased with increasing conformity.^30^ Comparing the results achieved within the present experimental study with those predicted computationally (Table 1), it is clear that they follow a similar trend of increasing wear with increasing conformity and higher wear with conventional polyethylene. Notably, there is higher agreement between the experimental and computational data for the higher wear rates, but there is a tendency for the experimental data to show higher wear rates than the computational predictions.

**Table 1.** Comparison of experimental wear rates with computationally predicted wear rates.

	Mean wear rate (mm^3^/Mc) (±95% confidence limits for experimental data)			
	GVF		XLK	
	Computational^30^	Experimental	Computational^30^	Experimental
Flat^21^	2.5	3.4 ± 0.7	0.6	2.3 ± 0.3
Lipped^31^	5.8	6.7 ± 1.5	1.9	4.0 ± 1.0
Curved^8^	8.7	9.2 ± 2.9	3.4	6.7 ± 1.5

Most recently, a combined computational and experimental study has examined the influence of contact pressure on the wear performance of a TKR.^32^ Notably, this was achieved through an increase in applied load and identical implants for each study. The increase in applied load resulted in both an increase in contact pressure and contact area, with a resultant rise in mean wear rate observed both experimentally and computationally. The authors highlighted a perceived discrepancy between their findings and previous simple geometry studies. However, it is important to note the difference in test conditions between their study and this study. In this study, the increase in contact pressure is resultant from the change in bearing geometry and subsequent reduction in contact area. In the published study,^32^ there is no such geometric change; thus, the increased load causes both an increase in pressure and contact area. It is proposed that the increase in contact area is the main contributing factor to the increased surface wear observed within that study, and thus the apparent discrepancy with this study.

Reducing contact stress to address delamination and mechanical failure of early generation, polyethylene was a key driver in fixed bearing knee replacement design late in the last century. However, several authors have highlighted the clinical limitations of using a highly conforming implant with over-constraint of the joint limiting natural motion of the knee joint and inducing additional stresses.^17,19,33^ Furthermore, lower tibio-femoral conformity has been identified in unicompartmental knee replacement design to enhance ‘natural’ rolling and sliding motion through the gait cycle.^34^

A recent retrospective clinical study, comparing a variety of different implants, including ultracongruent and cruciate-retaining fixed bearing knees has shown no significant difference in clinical outcome at a minimum follow-up of 10 years.^2^ It would certainly appear that the enhancements in polyethylene manufacture have enabled the re-visiting of less conforming knee replacement designs. However, it is important to consider conformity and wear performance in conjunction with other aspects of knee replacement design. For example, an entirely flat tibial insert would provide no natural constraint to the knee during motion.^33^ This study was conducted under displacement control, and thus, the relative motion of the femur on the tibia was controlled. Previous studies, contrasting displacement and force control of the displacement and rotation axes have demonstrated differences in both wear rate and relative motion of the knee replacement.^35,36^ Studies have demonstrated more variability in kinematics under force-controlled conditions compared with displacement control.^35,37^ Some studies have observed higher wear rates under force control;^36^ however, others had demonstrated no significant difference.^37^ This study was conducted under displacement control to replicate the constraints provided by the soft tissues in vivo. It would be likely that the lack of constraint of a flat TKR in a clinical environment would permit large translation, an increased wear region and create an unstable knee. The authors therefore do not propose that entirely flat inserts are adopted for TKR, however, a reduced conformity design may be appropriate for a partial knee replacement.^38^

This study has focussed upon the total gravimetric wear of the fixed bearing insert and assumes the majority of the volumetric wear to occur at the femoral-insert articulation. A number of studies have explored the influence of implant congruency on backside wear. A retrieval study highlighted more evidence of backside wear on implants with a higher degree of conformity, proposing the design enabled a higher transmission of torque to the insert–tray interface.^39^ Wasielewski^40^ proposed that lower conformity cruciate–retaining implants would demonstrate less backside wear compared with either posterior-stabilised or highly constrained implants, as the reduction in constraint would limit the transmission of shear force to the inferior insert surface. Therefore, there may be a twofold design advantage of the flat insert within this experimental study – a reduction in superior surface wear due to the changes in local contact area and a reduction in torque at the tray–insert interface, also reducing backside wear. However, experimental studies have also demonstrated backside wear to be highly dependent on the tibial tray design, and this study has examined only one such interface.^41^

**Figure 5.**
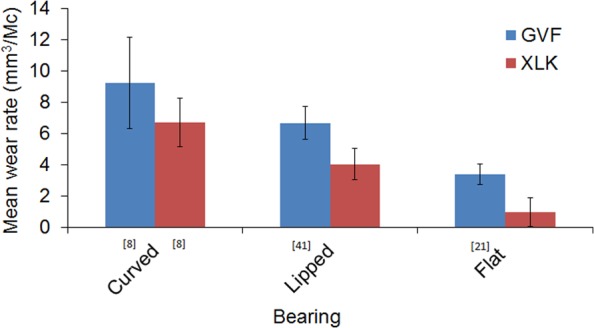
Mean wear rates for conventional and moderately cross-linked polyethylene for three different insert designs (source citation indicated).

This study explored the influence of conformity on the wear of a fixed bearing TKR using a moderately cross-linked polyethylene material. Combining this study with previously reported data, Figure 5 demonstrates the wear of the XLK material to be significantly lower than the GVF material in all test configurations.^8,21,31^ It has been proposed that cross-linking reduces chain mobility, making it more resistant to cross-shear forces than conventional polyethylene materials.^42^ Several other experimental studies have highlighted potential improvement in wear performance with cross-linked polyethylene in TKR, although many studies have examined polyethylene with a higher level of cross-linking.^43,44^ Studies have highlighted a reduction in mechanical properties with highly cross-linked polyethylene, and therefore a compromise between enhanced wear performance and mechanical properties must be considered.^12,13^

There appear to have been few clinical studies that have examined the performance of a moderately cross-linked polyethylene in TKR. A recent case study highlighted the repeat fracture of a moderately cross-linked tibial insert in one patient, at 16 months and 11 months following revision.^45^ Inspection of the inserts highlighted a number of subsurface fatigue cracks. The patient within this case study was reported to be severely obese; therefore, despite no unusual activity being reported, it is likely that the insert underwent high loading. Another recent prospective study has compared clinical performance of Sigma TKRs (as used within this study) with XLK and GVF inserts.^46^ At a minimum follow-up of 5 years, with a cohort of more than 400 patients, there has been no observed difference in the clinical performance of the two bearings, and, importantly, no evidence of mechanical failure in either group. A retrieval analysis compared highly cross-linked and conventional polyethylene inserts for bearing wear and levels of oxidation, finding no statistically difference between the two bearing types.^47^ To date, the improvement in wear performance observed experimentally does not appear to be reflected in the clinical environment, but there are still few longer term follow-up studies.

The oxidative stability of polyethylene has been widely investigated, and improved sterilisation processes have considerably reduced oxidation with respect to earlier generations of polyethylene. Recent studies have highlighted the potential for a relationship between the in vivo stresses experienced by a polyethylene bearing and the level of oxidation. Regis et al.^48^ identified this relationship through analysis of conventional polyethylene acetabular cups, and this finding was further supported by Kop et al.^49^ who showed a correlation between stress and oxidation in knee inserts, identifying the areas likely exposed to higher in vivo stress, such as the medial region to exhibit higher oxidation of the polyethylene. The authors also proposed that a stress-induced oxidation ageing process may be more beneficial than shelf-ageing to predict oxidative stability of a design and material. It is important to reflect on these findings when considering the results of this study, as the lower conformity bearings will elevate the contact stress.

Further examination of the whole dataset (Figure 5) allows comparison of both material and conformity. It is interesting to note that the wear rate of the flat conventional polyethylene is comparable to the lipped cross-linked insert, and likewise, the lipped conventional insert wear is similar to the wear rate of the XLK curved design. This suggests that there may be different approaches to achieving desirable wear performance – based on both material and bearing design. When considering the trade-off between oxidative stability, wear performance and material toughness of the material,^13,14^ the consideration of conformity, in conjunction with material selection potentially provides different solutions.

## Conclusion

This study has investigated the influence of bearing conformity on the in vitro wear of moderately cross-linked fixed bearing TKRs. This study has demonstrated that under in vitro kinematic conditions, there was a significant reduction in wear rate with decreasing conformity, and this effect was demonstrated more clearly when combined with previously reported data under comparable conditions. These findings, alongside improvements in material properties, highlight the potential for improved wear performance through conformity design; however, the authors note the need to balance the requirements of wear performance with the clinical requirements for good stability and kinematics.
